# Responding to health literacy of refugees in Australian primary health care settings: a qualitative study of barriers and potential solutions

**DOI:** 10.1186/s12913-024-11192-9

**Published:** 2024-06-21

**Authors:** Prince Peprah, Jane Lloyd, Mark Harris

**Affiliations:** 1 Social Policy Research Centre, University of New South Wales, Sydney, NSW 2052 Australia; 2 Centre for Primary Health Care and Equity, University of New South Wales, Sydney, NSW 2052 Australia; 3 Australia’s National Research Organisation for Women’s Safety, Sydney, NSW 1230 Australia

**Keywords:** Health literacy, Culture, Organisational health literacy, Barriers, Primary health care, Refugees, Australia

## Abstract

**Background:**

Organisational health literacy is a promising area of research that enables a focus on how systems and services can be designed in ways that are responsive to populations with varying states and levels of health literacy, knowledge, and practices, including African refugees. The challenge is how organisations and professionals do this in practice, and research in this area is in its early stages. This qualitative study examined barriers to implementing health literacy responsive care practices in primary health care settings in Australia. It also offered suggestions to potentially address the barriers to improving organisational health literacy.

**Methods:**

Refugees (*n* = 19), primary health care professionals (*n* = 14), and other key stakeholders (*n* = 19) were recruited through convenience and snowball strategies from three states in Australia: New South Wales, Victoria, and Queensland. All but one participant was interviewed face-to-face via Zoom. Semi-structured interview guides were used to guide the conversations. Transcriptions from audio recordings were analysed using directed content analysis.

**Results:**

Thirteen themes were extracted from the data. Themes were organised into the following categories: structural and systemic, organisational context, individual professional level, individual patient level, and socio-community level. Major structural and system-level factors affecting organisational health literacy included rigid systems and structures and limited time. Key organisation-level factors included inflexible organisational processes and policies, institutionalised othering, discrimination and racism, and lack of interpreters. Individual professional factors were poor communication with patients and cultural knowledge gaps. Linguistic issues and service mistrust were key individual patient-level factors. Socio-community factors included limited community engagement. Participants identified potential solutions to help services navigate out of the barriers and improve their response to health literacy.

**Conclusion:**

The findings suggest that mainstream services and organisations could improve timely and appropriate health care access and utilisation for refugees through strategies such as designing services and health literacy programs with refugee communities, promoting health literacy champions in the workforce, integrating health literacy and culturally responsive care plans and strategies into organisational priorities.

**Supplementary Information:**

The online version contains supplementary material available at 10.1186/s12913-024-11192-9.

## Background

Understanding the barriers to organisational response to patients’ health literacy is crucial for planning and designing services that are easy to use and appropriate to needs and preferences [1]. Actions to support organisational health literacy are defined as the extent to which health organisations design, establish, and implement policies, practices, and systems that accommodate needs and make it easy for all people, including refugees with different health literacy and knowledge states and levels, to easily access, navigate, comprehend, and utilise health services and information to improve their health and well-being [2, 3].

Accessing high quality primary health care, especially for marginalised populations such as refugees, is exceptionally challenging. Several structural and social factors impede this goal, including cultural differences, language barriers, structural racism, othering and discrimination, and health-system complexity [4–8]. Difficulty accessing health care is continuously exacerbated by systems and service demands [9]. When a health system consistently demands navigation, literacy, and other skills, knowledge, and abilities that marginalised persons, such as refugees in Australia, do not possess [3, 10], it systematically disadvantages them in terms of health care access [11, 12]. Thus, focusing on individual patients’ health literacy alone can prove burdensome because, it, ultimately places the responsibility on them to navigate and access health care services and information [13, 14].

Efforts are being made to improve the health literacy of organisations in Australia due to its importance to promoting patients’ access to health care [15–17]. For instance, Federal and State governments and entities, such as the Australian Commission on Safety and Quality in Health Care, have consistently called for a prioritisation of health literacy practices and research that focuses on eliminating barriers to navigating, accessing, understanding, and using health services [15]. The NSW Health Literacy Framework 2019–2024 also acknowledged the need to promote systems and workforces to provide services that meet refugee patients’ health literacy and cultural and linguistic backgrounds [18]. Moreover, scholars such as Trezona and colleagues have developed and validated a tool called the Organisational Health literacy Responsiveness (Org-HLR) for organisations to assess, action on and improve their health literacy [3, 19]. These health literacy frameworks and tools identify enabling factors for organisational health literacy. These include community engagement and partnership, community/peer navigators, interpreter use, family involvement in care, effective communication, cultural competence, easy and accessible health care environments, and leadership buy-in and support [3, 18]. Thus, these frameworks and tools aim to improve the fit between the health system and services and the needs of marginalised groups, such as refugees.

Overall, achieving organisational health literacy is an important idea that has not been systematically implemented across the health care system [20, 21]. Health literacy frameworks may be developed, but they are not routinely applied or adequate in scope to responding to the health literacy issues of marginalised populations such as refugees. Some reviews have shown that the barriers to system and organisational health literacy are universal [22–24]. They include poor communication skills of health professionals, lack of training regarding health literacy responsiveness, limited human resource capacity, lack of organisational leadership support, and low priority and commitment towards health literacy. Other organisational level factors include design and implementation issues, such as the absence of change champions, lack of a culture of change, the complexity of health literacy tools and guides, and lack of physical resources [22–25]. Some recent studies also reported facilitators such as established partnerships with external organisations, experienced and skilled staff, and active initiatives in clinical settings [1, 17, 25].

The development and testing of organisational health literacy frameworks, self-assessment tools and guides lead to insight about organisational performance, however implementation is often complex and difficult in practice [17, 20, 24, 25]. At the same time, there is limited evidence on the factors that either promote or hinder the application and implementation of these frameworks and tools in practice, especially in primary health care settings in Australia. Currently, one qualitative study in Australia explored the issues faced by General Practitioners (GPs) when supporting patients with limited health literacy and the strategies they used to support patients [26]. One pilot study using sequential mixed method approach also examined the efficacy of the Org-HLR tool and associated assessment process in a remote primary health care setting [25]. Another evaluation study based on a literature review established a case for the importance of appraising the health literacy of health care services in Australia [17]. However, these studies only involved clinicians and administrative staff and as a result, service users’ perspective on organisational response to health literacy remains largely unknown. Also, less emphasis has been placed on organisational response to health literacy of marginalised groups, especially African refugees who may come from different health systems with varying health literacy issues, knowledge, beliefs, and practices that affect their access to services [10, 27].

Here, these study addressed a significant knowledge gap by investigating barriers to organisational health literacy of refugees within primary health care settings in Australia. The study also investigated what can be done by health services to better meet the health literacy, knowledge, and practices of refugee patients. A qualitative study conducted from multiple perspectives at the primary health care level on barriers to organisational health literacy in Australia is needed to offer evidence to guide health organisations and decision makers in designing and implementing health literacy responsive care strategies and programs.

## Methods

### Study approach and population

This exploratory qualitative paper presents data on barriers to responding to health literacy of refugees among primary health care organisations and professionals. This study also offers evidence on how barriers can be addressed to improve organisational response to health literacy. The participants in this study included 14 primary health care providers (with diverse professional and disciplinary backgrounds such as GPs (*n* = 4), registered nurses (*n* = 5), nurse practitioners (*n* = 2), pharmacist (*n* = 1), paediatrician (*n* = 1), and psychologist (*n* = 1)) and 19 stakeholders (from different backgrounds such as health service directors (*n* = 4) practice managers (*n* = 3), multicultural health workers (*n* = 3), resettlement workers (*n* = 3), liaison officers (*n* = 4), and community elders (*n*= 2) from several local health districts and primary health networks across three Australian states: New South Wales, Victoria, and Queensland. Also, 19 refugees including both males and females from nine African countries also participated in this study. Details on the study design/approach, sample, and selection process, including inclusion and exclusion criteria have been reported in an earlier study [28].

### Recruitment

The South-Western Sydney Local Health District Human Research Ethics Committee approved the research (Ethics Approval Number: 2021/ETH11161). Informed consent was obtained from all subjects who participated in this study. Detailed report on the recruitment procedure(s) have been reported in a previous study [28]. Briefly, participants were invited to participate in face-to-face semi-structured interviews to offer their perspectives on factors that obstruct health literacy responsive care practices and strategies through different sampling techniques, including convenience and snowball strategies. Invitation letters containing flyers and information sheets were sent to primary health care professionals and stakeholders through professional bodies and organisations working with refugees. The flyers were also given to government and non-governmental agencies that either directly or indirectly provided services and support to refugees. Emails, text messages, or phone calls were used for follow-up. All participants who showed an interest in the study were screened to ensure that they were eligible to participate. The initial interview participants were asked to invite others within their networks to participate in the study.

### Semi-structured interview design

Three semi-structured interview guides were developed for providers, refugees, and other key stakeholders, respectively (see supplementary files 1–3). The guides were informed by and refined through literature review [19, 26], reflective supervision, and feedback from pilot interviews. A semi-structured approach was adopted because an understanding of organisational response to health literacy, especially within the primary health care context in Australia, is a relatively new area of research [29]. Data for this study were mainly gathered from two main open-ended questions that were asked to provider and stakeholder participants followed by a range of prompts that encouraged participants to reflect and share their experiences on 1) barriers to health literacy responsive care strategies, programs, and policies,and 2) perspectives on what can be done to address the perceived barriers to health literacy responsive care strategies, programs, and policies. Refugees’ perspectives on barriers and solutions to implementing health literacy responsive care strategies, programs, and policies were drawn from their experiences of primary health care access.

The first author conducted the interviews in English using an institutional Zoom platform, except for one stakeholder interview. The first author is an international student from Africa who has previously conducted extensive qualitative interviews with marginalised populations. However, he had no relationship with any of the participants before the interview. The interviewer understands how it is to live in an African country like Ghana and a Western nation like Australia. This knowledge enhanced his ability to conduct the interviews. More importantly, the research team composed of qualitative researchers with diverse knowledge and experience in conducting health services research, especially among service providers and marginalised health care consumers such as refugees in Australia. The interviews were conducted between March 2022 and December 2022. Data collection was stopped after the realisation that thematic saturation has been met [30]. Each interview lasted between 30 and 60 min and was audio recorded. Before the interviews, information sheets and consent forms were provided to the participants to enable them to understand the study and their participation before consenting. Refugees were given a $30 gift voucher for their time.

### Analysis

The first author and an external professional transcription company transcribed the interviews. The first author verified all transcripts for accuracy once received from the transcription company by listening to the recordings and comparing them to the written transcripts. The analysis involved a recursive process of several stages for key concepts in NVivo 12. Coding was conducted by the first author but was discussed with the supervision team. Revisions were made by considering emergent themes and interpretations. The directed content analytical approach guided the analysis of patterns and themes within the data [31]. Directed content analysis is a flexible content analysis approach commonly used in health care and service research to interpret meaning from the content of text data when the structure of the analysis is operationalised based on an existing theory/model or prior knowledge on the topic under investigation [30–33]. The main goal of the approach is to test, correct, and/or possibly extend and enrich an existing knowledge or a model unlike the conventional content analysis approach which seeks to derive new theories [30, 33, 34]. Thus, the directed content analysis was selected over the conventional approach because of its flexibility and ability to extend existing knowledge and model. In this study, the approach was used to guide, examine, and extend knowledge and models regarding organisational health literacy in the context of refugee population to enrich existing evidence and understanding.

The analysis identified two key themes: 1) the barriers to health literacy responsive service strategies, programs, and policies; and 2) solutions to the barriers. Data were categorised as a barrier if, according to the participants, the factor(s) made it difficult or impossible for organisations and professionals to respond to the health literacy needs of refugee patients and communities. Next, we went to the dataset for a more intensive analysis and identified codes and placed them under their respective categories along with key quotes. Through this iterative process, involving going back and forth to the dataset to obtain further evidence, further codes were developed and eventually collated into themes. When there were differences in opinions among the research team about the codes and themes, consensus was reached through further discussions.

Thematic categories were created based on the observed patterns of meaning in the dataset, along with solutions to navigate out of the barriers. Finally, since the development of the initial interview questions was guided by the literature [3, 19] the directed content analysis results/categories were organised based on five levels derived from the literature applying systems theory to health care systems. These levels included structural and system levels (the broader external Australian health system), organisational context, individual professional level, individual patient level, and social community context.

## Findings

### Barriers to health literacy responsive strategies, policies and programs

**Fig. 1:**
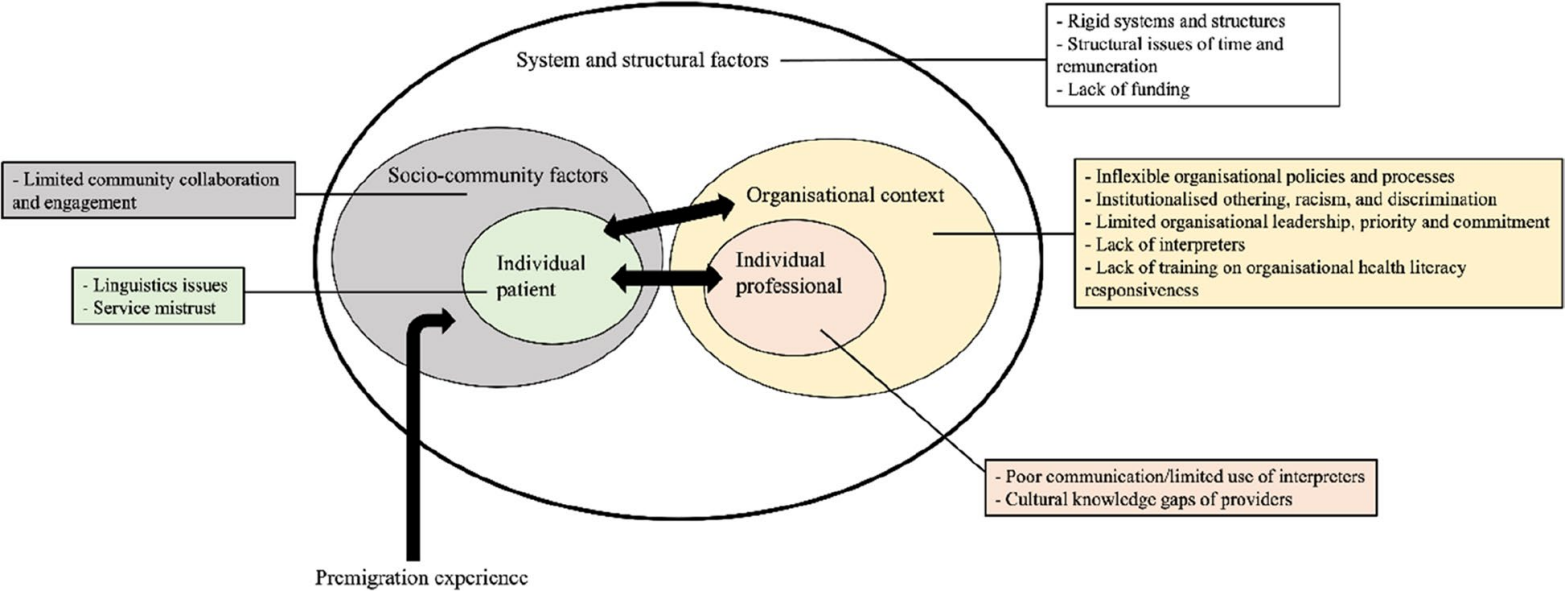
Summary of the thematic categories interacting with each other

The following section describes the themes which are organised into 5 topics. The section includes a diagram summarising the themes and how they interact with each other (see Fig. 1).

#### Structures and systems (the broader external Australian health system)

Structural and system-level factors were grouped into three categories: the structural arrangement of the broader Australian health system, structural issues of time and remuneration, and lack of funding. Providers and stakeholders perceived the traditional/hierarchical structure of the Australian health care system that delineates roles, responsibilities, and relationships among health care organisations as rigid. Provider and stakeholder participants mentioned that the structure of the health system significantly influences the care coordination, delivery of quality services, and patient health outcomes such as access and health literacy. The provider and stakeholder participants stated that the Australian health system’s hierarchical structure impedes changes and innovation, mainly due to the changing health care environment.

*… the health system is rigid, the structures are rigid, and the organisations are rigid. So, it becomes difficult if you want to do something new because it is not easy to change the structures. I think it goes down to the rigid structures and systems that do not allow this improvement and change. [Nurse]*

One service director also lamented the hierarchical structure of the Australian health system by focusing on its rigidity and complexity that do not promote flexibility and patient-centred care.

*The system has it[s] several hierarchies and… culture which is very complicated and rigid to my view… everyone working there including doctors and services… are rigid [;] they are not flexible. [Service director]*

Some providers mentioned that, in most cases, organisations and professionals are motivated and willing to improve and adapt their practices to respond to the health literacy needs of marginalised groups such as refugees. However, the structural issues do not support them.

*… I don’t think we do a very good job in educating our clients unless we’re asked to [do so], and I really think it goes down to the structures and systems in place that are rigid to allow this education… we basically cannot do more enough to* [help] *those people… who do not have the capacity. [Nurse]*

One provider participant reflected in support of the above:

*… it is not easy to do sort of things because you are working with the broader bigger health system that is… too immovable so hospitals… clinics and people* [working] *there may want to do things differently… to help* [but may not be able to do so]*. [Nurse practitioner]*

The rigidity of the health care system left some providers feeling frustrated about the difficulties in generating change within the system.

[I see a lot of committed people]*… but time is not there because this takes a lot of time, this takes a lot of energy, it takes resources,* [but] *there’s no time… people who completely understand what is happening* [within the system]*, just get frustrated… [because] they’re not getting what they’re asking for to change things, and then they just get frustrated…* [and] *burn out. [Multicultural health worker]*

Time was portrayed as a critical structural hurdle in most interviews among providers. For instance, providers mentioned that using interpreters and models, such as teach-back, requires time, and formal consultation or appointment cannot be sufficient.

*…the structures are so rigid that they dictate the duration of the consultation. So, you see a refugee patient for 10-15 minutes, which is never enough to support them in building their knowledge levels. Sometimes, you wish to help, but the time is not there. [GP]*

Some providers also focused on systems that did not allow for interpreter use because of the structural issue of time.

*Getting them to use interpreters is one of the biggest problems, and… getting them* [to] *take their time to let people understand things better is another thing… I think it is because of time because within 5 minutes you should leave the office for someone to come in and I think it is because of the system and structures we have… [Nurse]*

Some providers also re-emphasised the structural issue of time, focusing on the effect of time on culturally responsive service delivery in mainstream services.

*The main challenge* [is] *that* [it] *obviously takes longer, it’s more resource intensive, and that’s the very reason why you’ll find so many like GPs or health services like hospitals*… [not doing that]*. It takes too long, and they have too many people to see… [Nurse]*

It was mentioned that delivering culturally responsive care for patients from refugee backgrounds requires time to build rapport and trustful relationships.

*I guess time is [an] important element because doing things in a delicate way [is important] so that you do not rule out… [people’s] norms… practices [and] beliefs… [It] takes time… building rapport and knowing them and… it is one reason mainstream services cannot respond to these people well because they do not have time. [Nurse]*

Providers explained that seeing a patient from a refugee background is a double appointment since they require longer consultations and extra-consultation activities that cannot be paid or remunerated under the current Medicare billing system.

*I think the workload as well… In fact, not just the time but… [also] the pain [because] you get no extra pay. Sometimes you wish to help but end up sticking with your regular appointment. Using phone interpreters… sometimes… takes a good three [to] five minutes to get the phone interpreter working in the consultation… So, every refugee patient I see… is a double appointment, and you don’t get paid for that and [that is] … hard… [GP]*

External funding is needed by organisations to effectively respond to the health literacy and cultural needs of their service users and broader communities. Providers and stakeholders mentioned that funding for community activities, consultations, programs, and education could help address health literacy issues among culturally and linguistically diverse communities such as refugees. Nevertheless, stakeholders and providers predominantly mentioned funding. They explained that they have limited financial resources and capacity to undertake community-based programs, education and activities that foster and sustain health literacy among marginalised communities.

*How we can get the finances… that’s the big[gest] issue… I’m getting the government to recognise it… I think we do [a] great work, but we do not have the funding capacity, and no one is helping us. [Multicultural health/resettlement worker ]*

For instance, some stakeholders shared that some health literacy promotion activities and programs within communities by their organisations could not be continued due to a lack of funding.

*… the fact is that we do not have the needed funding for our work most of the time… there was a great piece of work done around the Afghan community… we need to do that for a whole lot of backgrounds, but we just don’t have the capacity… funding capacity. [Service director/manager]*

The above description suggests that structural barriers to providing refugees with health-literate and culturally responsive care may not be new. However, more interesting is the acceptance of the system’s limitations by providers and other stakeholders. In addition, the system’s inadequacies must be overcome by the goodwill and discretion of the individual providers. For instance, providers had to see a patient for a double appointment, even though they were only charged for one consultation.

#### Organisational context

In this context, five categories were identified from the data: 1) inflexible organisational policies and processes; 2) institutionalised othering, racism, and discrimination; 3) limited organisational leadership, priority and commitment; 4) lack of interpreters; and 5) lack of training. Provider and stakeholder participants felt that policies and processes, within many primary health care organisations do not support health literacy development because they are inflexible.

*… for* [all] *this while, I understand primary care need to be very flexible, but I doubt if that is what we see… in Australia, because most [of the] things like policies… processes and systems in the organisations are not flexible... It cannot be change*[d] *or modify so easily to make change occur and I guess because the general practice itself is rigid* [and] *the health system is rigid. [Nurse ]*

Some provider and stakeholder participants lamented the inflexibility of actions particularly within hospital services such as communicating health information to patients, utilising medical and non-medical approaches to perform wide range of patient care education activities, and contacting patients for care. For instance, one stakeholder participant was concerned about how hospitals contact or share information with patients:

*… when they… send out appointments, they’ll send it out in English to the person* [and when the]*… person doesn’t respond, they take them off the waiting list. So that’s an example of a… culturally unfriendly and unresponsive service because people get stuff in the mail in English all the time. And if they don’t speak English, you can’t tell that, it’s ridiculous. [Service manager]*

Due to the perceived inflexibilities within services, this participant maintained that it was difficult for organisations to adopt a right-based approach to service delivery by adopting an individualised approach to care.

*… it’s really hard to… take a rights-based approach, which I think should be taken. People have got a right to… equal service... and if you’re looking at hard to reach communities and you have to reach out, it’s not okay to just use the same system or follow up… when services are asked to do things differently it becomes a problem. [Service director]*

Some providers stated that the inflexible processes have created a one-size-fits-all approach or model for service delivery to every patient, including refugees.

*…we provide a generalist service without special consideration to people from different backgrounds with unique challenges and needs. [Nurse]*

All participant groups stressed various forms of discrimination and racism within health institutions as barriers to responding to health literacy of refugees. Refugee patients face racist and discriminatory attitudes and behaviours in mainstream health services. A notable description of these accounts is the concepts of blackness and othering, especially in mainstream primary health care. One stakeholder reflected on the blackness below:

*I think the biggest barrier is ourselves, and ourselves I mean the structures, the providers who are whites, the structures that are for the whites… we assume that everyone gets the same level of… education… [as] what we do. How we see people who are not natives as ‘others’, especially those who are black. So, more often, we use the words like ‘others’ and ‘blacks’… it is rooted in how we perceive people, greatly affecting how we interact, engage and support people as professionals. Because ‘Others’ are out, if you are ‘black’, you are out. Everything, Anglo. We’re very naive and ignorant population. Generally, we also see things through a very Anglo prism. [Service director/manager]*

According to some providers, the Anglo worldview mentioned above has shaped health professionals’ and organisations’ attitudes, processes, and practices, especially within mainstream services.

*Probably structural racism or institutional racism.* [There are] *so many awesome clinicians that we work with that are great. In the mainstream hospitals… unfortunately,* … [some of the nurses and doctors] *have absolutely no idea how the world works outside of their very small country. Despite they’re not outwardly out here, obviously, [they go] … about their day trying to be horrible and racist or whatever… and in their actions… [and] … they show that there is a long way to go… [Nurse]*

One participant from a refugee background shared the following experiences:

[I think] *some health professionals… are racist because I when* [I] *came I remember that I saw one professional… and he wanted me to speak English and no interpreter too* [was available] *so he said we are tired and fed up with all the refugees coming here and can’t speak English. Why are they here if they can’t speak English in the first place. In fact, I was very sad the whole day. He did not know our experience. [Refugee]*

One provider participant from a refugee-focused health service envisaged a future in which services would be responsive and less racist:

*I would like to see a time in the future when we do not have to do this work [referring to refugee-focused services] because the…* [hospital] *is just better and more responsive and less racist and less Anglo-centric. But good luck to me. [Nurse ]*

Organisational health literacy frameworks have stressed the importance of organisational leadership support and priorities. However, for instance, participants collectively lamented that health literacy was not an organisational priority.

*I think the commitment is not there* [and] *the leadership… do not see health literacy of people like… as a big thing because when the leaders decide to build health literacy, it becomes important priorities for them…* [and] *you can see that in their plans, procedures, and protocols… [ Community liaison officer]*

Lack of interpreters was also mentioned as a barrier to responding to health literacy among refugee, provider, and stakeholder participants. Refugees, stakeholders, and providers mentioned that there are minor and emerging languages in which it is difficult to find interpreters.

*When it comes to translation, they have materials in other languages, but the problem is the minor ones; they miss out and there are some that do not have interpreters at all which is one of the issues in trying to reach the vulnerable. [Refugee]*

Some stakeholder participants confirmed the following:

*... recently, I had a request for a language by one psychiatrist that I never heard in my entire life. We couldn’t find interpreter so we couldn’t assist the patient according to the policy. Policy says, you have to organise professional interpreter,* [but] *we didn’t have… so we had to ask family member to assist, which was not* [a] *good practise, but there was no simply other choice because there is no such a language registered in Australia. [Multicultural health worker]*

Stakeholder participants specifically discussed that lack of or limited formal education and training on culture and mandatory cultural training for health providers and administrators are other barriers to culturally responsive care.

*I think a big challenge is that… it’s not something that… they get formal training in… I think they do not have formal training in cultural stuff… training about [cultural] awareness for refugee communities was not mandatory. So, it could be tricky to get a group together. And it was really based on whether the manager of the service was interested and passionate about it and then they would get us in. [Community elder]*

The participant further supported the importance of cultural training in working with refugees.

*For example, doctors will come from North Shore to work in Blacktown and where a vast, diverse population from all different backgrounds exists. They have not really experienced that, and they do not know what to do because they have grown up in a completely white neighbourhood and do not have that formal training for cultural inclusiveness. [Multicultural health worker]*

The description of the above five categories in the organisational context shows some interconnections. For instance, accepting the rigid system and its inadequacies indicates that for change to occur, health literacy has to be an organisational priority to drive change. If responding to health literacy is not a priority, it is left to the goodwill and empathy of providers to tailor care to patients’ needs (despite the system’s limitations).

#### Individual professional

Poor communication/limited use of interpreters with refugee patients and cultural knowledge gaps of providers were also identified at this level. Refugee participants expressed that there were always assumptions in communication and information sharing by health organisations and professionals.

*One issue is that most of the GPs think everyone can… speak English and they do not like using interpreters, they do not have time to see if we have understood things, so many go out and they do not understand anything…. [Refugee]*

The quote suggests inherent assumptions and generalisations in language services, such as the assumption that all patients could communicate in English, which is likely to prevent refugees from finding and understanding health information.

Providers’ limited use of interpreting services, especially in mainstream services, emerged as a converging concern and a critical barrier to primary health care organisations and providers’ response to health literacy. Language differences create the issue of provider-patient communication discordance.

*So, we were interacting with the bigger health system, and it is still a struggle to get people to use interpreters to provide language support to this population. It is a fundamental requirement, but it is a serious challenge. This challenge is common in mainstream services… it is a huge barrier. [Service director/manager]*

Knowledge gaps regarding understanding refugees’ cultural backgrounds, identities, and needs among individual health professionals were specifically mentioned in the interviews as barriers to culturally responsive care delivery. Stakeholders and refugees mentioned that many professionals did not have knowledge and experience working with cultural groups, such as Africans. Stakeholders and refugees specifically stated that many providers working with refugees have no knowledge about specific cultural belief systems and practices of refugees that shape their health and health care decisions. Again, they emphasised that many providers need to better understand how to engage people like African refugees who are culturally diverse, sensitive and have experienced significant trauma and loss.

*…I think there are instances in which they [referring to providers] are mot culturally appropriate… [because] they tend to put all Africans in one box. So, what Somalians want… they think Sudanese also want because [we are] dark skinned. They don’t try to find out which part you are coming from, your beliefs, and all of that… they just group us together and say we are all the same. [Refugee]*

Due to the perceived knowledge gaps, one stakeholder participant stressed that many providers need a rulebook regarding how to deal with specific cultural groups.

*… it always comes back to not giving a rule. People want a rule book. This is how I deal with Ghanaians… this is how I deal with Kenyans… but being prepared to put themselves out of* [their] *beds and to understand that there are differences… and things aren’t necessarily going to be the same… [Multicultural health/resettlement worker]*

Another stakeholder participant added the following:

*So, some people think that there is a way to talk or to engage people with [refugee backgrounds] … they want to know the formula and really, there’s no formula. [Multicultural health/resettlement worker]*

Refugees and stakeholders highlighted that most providers sometimes lack experience, skills, and confidence to communicate health information in a culturally sensitive manner. Again, they stressed that some providers were unaware of refugee patients’ cultural background and identity.

*But look at the person who hardly knows any cultural background, who hardly understands where you’re coming from, who thinks that… people in the hospitals even struggle to engage [us] because they do not know [our] cultural issues to be able to understand [the] issues [we are facing]. [Refugee]*

The above quotes imply the need for cultural competency, awareness skills, and training of providers. Training and skills will help build knowledge and confidence in engaging and communicating effectively and appropriately with refugee patients. Effective engagement and communication are essential for health literacy development and culturally responsive care.

#### Individual patient

Linguistics issues and service mistrust among refugees were reported at this level. The providers mentioned that most refugees had limited English proficiency in communicating effectively with providers who did not speak their native language. Refugees stressed that health literacy and cultural concerns in interpreting services are often ignored. Refugee participants argued that providers and organisations cannot assume that interpreters share the same culture with refugee patients because they speak the same language. Several dialects exist within the same language and interpreting services do not consider them.

*… but for us Sudanese there’s another problem… there were no Sudanese interpreters, there was no African interpreters, so we were serviced by some other Arab nationalities… like Lebanese or Iraqis… [but] … there’s a difference also in the dialect… [Refugee]*

The participant also talked about differences in English accents as a challenge in communication.

*… We have our own African language, we have the Arabic language, which is the language of our country, Sudan, the general language, so English was not that familiar for ordinary people… unless you … [you go] to school… but… the accent here is very different. [Refugee]*

Both providers and stakeholders alluded to mistrust as a barrier to organisational response to health literacy. It was revealed that most vulnerable groups, including refugees do not trust health services and professionals because of past experiences in their home countries and Australia. Providers felt that they needed people’s trust to help them build their health literacy and respond to their cultural needs, but when patients do not trust, the services and professionals’ engagement become compromised. Additionally, patients become less receptive to health information if they do not trust the system or professionals.

*… trust is important… because they have lost trust before coming here. I remember some clients used to visit our services and they stopped, and I guess because they did not trust [us] because whenever I gave them some information and explained some medical issues, I noticed they did not believe or trust me. I think some of my colleagues here faced the same sometimes. [GP]*

The factors discussed by the participants at the individual patient level showed some interrelationships between them. For example, linguistic issues may create negative experiences which could impact the trust people have in the services and professionals.

#### Socio-community context

One category was identified at this level based on the data: limited community collaboration and engagement. Refugees, community leaders, and elders expected opportunities for partnerships, collaborations, and the co-design of interventions to ensure that services meet their health knowledge and practices. Stakeholders lamented that health professionals and organisations operate a top-down approach and often fail to consult community elders, leaders, influential people, and community-based organisations in planning and designing health literacy activities and programs, creating a gap between services and communities.

*The fact is that they failed to consult, in fact… they always fail to consult people like us, multicultural workers* [and] *community intermediaries. They mostly do what they want first and when things get worse, then they try to consult not realising that we work with [the community people] and they understand us more than them. [Multicultural health/resettlement worker]*

The limited involvement of refugee communities may mean limited access to services by communities as many refugees consult community leaders for health information.

### Navigating out of the barriers – suggested potential solutions

Participants were asked what should be done to address the barriers in implementing health literacy responsive policies, programs, and strategies; improve organisational response to health literacy; and help the refugee community navigate and access services that they need. Their suggested solutions are summarised into some key points in Table 1.

**Table 1:** Suggested potential strategies for addressing the barriers

Mandatory and culturally sensitive language support services
Making changes in resettlement/on-arrival policies and programs
Integrating health literacy into Federal/State level and organisational level plans and priorities
Modifying usual practices within organisations to promote tailored and targeted services
Employing more peer health navigators and bilingual community educators
Consumer representatives
Understanding the demographics and identities of patients from refugee backgrounds
Mandatory cultural awareness and competency training and education
Consulting and co-designing of health literacy and culturally responsive services and interventions with refugee communities
Taking community development approach to health literacy responsiveness

## Discussion

This study aimed to explore the structural, systemic, organisational, personal, and community level factors that serve as barriers to organisational health literacy from the perspectives of primary health care services/organisations, professionals, and refugees. The study also identified potential solutions to address these barriers and improve service responsiveness for refugee populations in Australia. At the structural and system level, the study we found that the hierarchical structure of the broader health system which is perceived to be rigid, structural issues of time, and lack of funding and remuneration serve as barriers to implementing health literacy responsive policies, programs, and interventions. These factors indicate the importance of external structures, systems, and policies in organisational health literacy [3, 11, 26].

The findings further extend the literature on and challenge many organisational health literacy conceptualisations that recognise only organisational responsibility and context factors and neglect the impacts of external forces, such as broader health systems and structures and the role of external bodies, such as governments. Health system structures and policies are critically important, but are uncounted for most health literacy frameworks and self-assessments [2, 3, 19, 35]. For instance, the Org-HLR [3] and the ten attributes of health literate health care organisation [2], which are popular organisational health literacy responsiveness models do not specifically consider the influence of the structure of the broader health care system on how organisations respond to patients’ health literacy. Although health organisations can implement policies and strategies to respond to health literacy issues and promote equitable access to health care and services, health literacy issues cannot be fixed exclusively at the health organisation level. Health organisations interact with and are produced and shaped by broader health system arrangements outside health care organisations [3, 36]. The findings, therefore, suggests that organisational health literacy frameworks/models should consider other relevant levels such as the health care system to promote effective systemic and comprehensive response to patients’ health literacy.

The findings indicated that organisations needed external funding to implement health literacy responsive care interventions and projects within communities. Previous evidence suggests that becoming health literacy responsive is resource intensive because more programs, changes, and staff are required simultaneously within an organisation. This finding also supports Trezona et al. [3] organisational health literacy framework. The external funding environment shapes organisational health literacy (which is not within the organisation’s direct control), such as the role of governments and other relevant funding bodies in providing adequate and sustainable funding for implementing health literacy responsive care programs and policies within organisations [25]. Therefore, this finding demands health system policymakers to acknowledge that responding to health literacy of patients is not the responsibility of only health organisations but also requires well-structured and flexible health systems that provide reliable funding for health literacy promotion.

The findings emphasised the relevance of organisational context in responding to people’s health literacy issues, in support of previous studies [17, 19, 25]. Trezona et al. [3] stressed that an organisation’s policies, processes, and systems must be flexible and modifiable to ensure effective and responsive services, program planning, and delivery. In contrast, participants stressed that structures, policies, programs, and systems within primary health care organisations that are supposed to be flexible are relatively rigid, which does not allow for health literacy responsiveness shifts. These rigidities within primary health care organisations can be linked to the reported rigidity of the broader Australian health care system within which the organisations are located.

For instance, many professionals and stakeholders expressed concerns about their inability to respond to patients’ health literacy issues, especially marginalised ones with peculiar needs such as refugees. They attributed their inability to the complexities within the organisations resulting from the hierarchical and perceived rigid health care system in Australia. From this finding, for most health organisations, the transition to comprehensive health literacy responsiveness may be a complex, unfolding process over many years due to structural rigidities that do not allow for change and innovation [23, 37–39]. Health organisations need flexible structures, processes and policies to promote organisational capability and functioning regarding organisational health literacy [3].

Also at the organisational level, we found for the first time that institutionalised othering, racism and discrimination influence organisational health literacy. Health literacy and the cultural needs of marginalised groups, such as African refugees, cannot be separated from the bigger picture and framework within which health organisations and professionals operate [40]. All participant groups reported perceived institutionalised othering, discrimination, and racism shape how organisations and professionals respond to health literacy of refugee patients. This finding can be linked to the reported racial, discriminatory, and Anglo-centric structures of the Australian health care system [41]. The Anglo worldview narrated by the participants may shape the attitudes, processes, and practices of health professionals and organisations, especially within the mainstream services that tend to favour others and discriminate against others regarding how they respond to and support different patients’ needs.

Moreover, the experiences of racism and discrimination, as highlighted by all participant groups, including health professionals, can also be linked to the broader African humanitarian resettlement in Australia, which remains a contested debate in public discourse, with conversations regarding increased levels of racism and discrimination [40, 42]. Evidence suggests that delivering equitable and appropriate health services to refugees cannot be separated from the politicisation and racialisation of resettlement across Australian political and public spaces [43]. Thus, interviews reflect and resonate with this evidence, viewpoints, and other previous studies reporting experiences of racism and discrimination in primary health care settings and organisations in Australia [40]. Thus, the finding implies that focusing on individual attitudes and actions alone may not be enough to address racism and discrimination within health services [41]. Organisational and policy level changes and interventions are needed owing to the perceived institutionalised nature of the racism reported in this study. Interventions such as health policy reforms, effective organisational antiracism policies design and evaluation, effective community and stakeholder engagement, antiracist education and professional training, and effective and evidential cultural competence and sensitivity training may help to address institutionalised racism within health care settings [4, 44, 45].

Based on reports of perceived institutionalised racism, it is not surprising that mistrust was reported as a barrier to health literacy responsiveness because the perceived racist and discriminatory attitudes of some services may affect trust in both health care providers and organisations among refugee patients [46]. Trust influences engagement and access to health systems, services, and health care providers. Among refugees, trust is a crucial factor that influences the extent to which they familiarise themselves with health systems, the amount of health information they can share with and receive from health organisations and providers, and the degree of power and autonomy they can exercise [46]. Thus, for health professionals to support patients’ health literacy, trust is essential because health information is more receptive when there is trust. Other organisational and individual professional-level barriers were found in this study, including organisational leadership priority, and commitment, linguistic issues, and poor communication. However, these factors have been extensively reported and discussed in earlier systematic reviews [22–24] and a recent qualitative study in Germany [1]. Their confirmation in the present study suggests their importance in organisational health literacy and the need for organisations to address them.

At the individual professional and patient levels, this study showed that cultural and racial-specific factors, such as cultural knowledge gaps shape organisational health literacy. This finding represents a new insight and an important research area and policy discussion within the health literacy literature offered by the present analysis. Many refugee participants and stakeholders mentioned that, in most cases and times, health professionals lack knowledge and awareness about refugees’ experiences, needs, backgrounds, and identities. They also lack experience, skills, and confidence in engaging, interacting, and communicating with cultural groups such as refugees, in a culturally sensitive and appropriate manner.

Organisational health literacy requires organisations and providers to provide services that meet all people’s cultural and health literacy abilities, needs, and preferences and support individuals and communities, such as providing language support and services for effective engagement and communication [3, 19, 25]. This finding particularly supports the interrelationships of culture, language, and health literacy, especially among refugees who are from culturally and linguistically diverse backgrounds like African refugees. Evidence suggests that health literacy can be pursued within the reality of an individual’s culture, and vice versa [47–49].

Another new finding was how limited consultation with community leaders, organisations, and gatekeepers affects the delivery of health literacy responsive services. Stakeholders mentioned that programs and projects promoting health literacy responsive care among communities operate in a top-down manner. Community organisations, elders, and leaders expect opportunities for collaborative programs and service design to ensure that services meet the needs of the people; however, such avenues are mostly not provided by services. Therefore, there is a gap between services and communities in terms of access to health information and messages. This finding, therefore, indicates that health services and organisations should actively engage and co-design health literacy interventions with the population that they serve as suggested by some of the participants. The importance of stakeholder and community engagement and co-design of health literacy programs and interventions would include a better understanding of community health literacy strengths and weaknesses, tailored health literacy and culturally responsive service programs, and community ownership of programs and projects [2, 3, 50].

### Moving forward: lessons reinforced and possible solutions

Collectively, the data provided by this study provide opportunities for improvement by organisations in terms of creating accessible, understandable, actionable, and useable health environments, information, and services. The study highlighted what is not working well, especially for refugees in terms of organisational health literacy. Participants listed potential solutions to address barriers and improve responsiveness and access to services, especially for refugees. The suggested solutions included prioritising language support services, such as the use of culturally appropriate interpreters and translators; consulting and co-designing organisational health literacy plans, services, and interventions with refugee patients and their communities; and prioritising and integrating health literacy and cultural factors into organisational plans and strategies. Other suggestions also included making changes in on-arrival policies, such as the orientation to allow for effective education about the health system, structural and system-level shifts that allow for flexibility for change, and modification of usual practices and policies to promote tailored services. In addition, providing a workforce with mandatory cultural awareness and competency training can help to address cultural and race-related barriers. In addition, employing peer health navigators and bilingual educators could be beneficial for easy navigation.

The next steps can be organisations taking and implementing these solutions to address the identified barriers found in this study, especially as most of the factors mentioned are modifiable. Regardless of how or where health organisations begin from, organisational health literacy is a critical tool for partnering with communities, families, and patients in pursuit of adequate access and health equity, especially for marginalised populations such as refugees. It is important for services to be aware that most refugee communities, families, and individual patients interact with health environments and information so that they can actively understand their health, access care, and make meaningful decisions. Thus, there is an added sense of moral and ethical urgency for organisations to act considering the observed racial, cultural, and ethnic inequities in health outcomes, which are to some extent caused by health system complexities and culturally insensitive care.

### Implications

Creating a health services and environment for patient to easily navigate, access, and use is a complex and multidimensional task. Identifying and examining the barriers to organisational health literacy provides a good understanding of what is working and what requires further improvement to promote health equity, especially for the refugee population. The expectation is that a refugee patient knowledge and practices, resources, and needs will meet the health system and organisational demands and requirements. This is vital because studies show that services do not meet the health literacy and cultural needs of culturally and linguistically diverse groups, including refugees [51].

Primary health care organisations and professionals must engage refugee communities, families, and patients in co-designing interventions to promote easy service navigation and access. Primary health care organisations should often conduct health literacy responsiveness assessments to develop more equitable processes, policies, and practices that can lead to adequate access to services to promote health equity. Tools such as the Org-HLR [3] and the ten attributes of health literate health care organisation [2] can help organisations to assess and improve their responsiveness. This is especially true for African refugees, given that they are from a completely different health system and resettled into fragmented complex health care environments in Australia, as well as many challenges faced by them in navigating and accessing services. Resettlement is a significant determinant of health as it affects individuals’ health literacy and health beliefs [11, 52]. It is, therefore, an essential, a moral, and a legal responsibility as enshrined in the human right to health [53, 54] for organisations to ensure access to care for refugees by improving their health literacy.

## Strengths and limitations

The study included multiple participant groups for diverse perspectives and insights on barriers to responding to health literacy at the primary health care level. This study adds value by offering concrete insights into addressing barriers to organisational health literacy. However, this study has some limitations in interpreting the findings. First, it focused on primary health care providers, stakeholders, and African refugees. It also focused on refugee patients who could communicate in English as part of the inclusion criteria. Further qualitative research involving other groups, such as the general population, refugees with no or limited English proficiency, health professionals in mainstream services, and hospital-based investigations, could be helpful. However, the conformity of the study findings with previous studies suggests that the present findings can be representative of other health care organisations in Australia and other settings with the same or similar health care systems and services. In addition, although the study findings were subjected to several discussions and comments, the results were not shared with participants.

## Conclusion

Removing barriers to service navigation, access, and utilisation, especially for marginalised populations such as refugees, is crucial for promoting health equity, but proves to be challenging for many health systems and organisations. This study represents the first research on primary health care providers, other key stakeholders, and refugees’ perspectives regarding the barriers to responding to health literacy. Overall, the directed content analysis revealed interconnected barriers to health literacy responsive strategies and policies. This study identified for the first time that cultural and racial-specific factors, such as cultural knowledge gaps and lack of cultural training, service mistrust, institutionalised othering, discrimination, and racism, shape organisational health literacy. This study yielded concrete strategies and recommendations for overcoming barriers and improving health literacy responsiveness. The findings suggest that services and organisations could improve timely and appropriate health care access and utilisation for refugees through strategies such as co-design of services, programs and interventions with refugee communities, culturally sensitive language support services, mandatory cultural awareness and competency training and education, and integrating health literacy responsive care plans and strategies into organisational priorities.

### Supplementary Information

Supplementary Material 1.

Supplementary Material 2.

Supplementary Material 3.

## Data Availability

No datasets were generated or analysed during the current study.

## Abbreviations

GP: General practitioner
HL: Health literacy
Org-HLR: Organisational health literacy responsiveness
PHC: Primary health care
