# Supplementary material for: Responding to health literacy of refugees in Australian primary health care settings: a qualitative study of barriers and potential solutions
Source: BMC Health Serv Res. 2024 Jun 21;24:757. doi: 10.1186/s12913-024-11192-9 (PMC11193211; doi:10.1186/s12913-024-11192-9)
Supplement: Supplementary file 1 — Supplementary Material 1. [file 12913_2024_11192_MOESM1_ESM.docx]

|  | **Interview Schedule** |
| --- | --- |
| ***Introduction*** | My name is Prince Peprah and I come from Ghana. I arrived in Australia in March 2020 (a week before the international travel ban) to do my PhD at UNSW—mix feelings and experiences due to COVID-19. Thank you very much for agreeing to participate in this interview. Sharing your experiences with us will help the researchers to understand how to support primary healthcare organizations to be more responsive to people from refugee background. We would like the interview to be a conversation, we really want to hear about your experiences accessing health care in Australia.  Please note:   - You can withdraw from the interview at any time. The interview is confidential   Are you happy for the interview to be audio-recorded? This will allow me to concentrate on the discussion and not focus on taking notes.  The interview will be transcribed and de-identified.  Do you have any questions before we proceed? |
| **Basic details** | 1. Can you please tell me a little about yourself?  (Prompts: Professional background, gender, age, educational attainment, country of origin, number of years spent in Australia) |
| **Research question 1** | ***To examine how primary health care organizations and providers identify and support health literacy needs of refugees from African nations*** |
| **Interview questions** | 2. Can you please tell me about your experiences when you visit your healthcare provider (**Hint**: provider here refers to General Practice doctor and nurse, community nurse, nurse practitioner)?  (Prompts: experiences about trouble seeking health care in Australia, how you found out what services to use, booking appointment, finding your way out, what involved in your care, how you understand the treatment and recommendations, how you and your provider communicate and interact with each other, how you used the information the provider gave you)  3. Do you believe when you visit your provider he/she understand/identify the problems you have mentioned such as………?  (Prompts: If yes, how the provider identifies the problems, how it makes you feel, how the provider supports you address the problems; if no can you please comment on why?)  4. Do you have any other comments about how providers should assist you get, understand and use information and health care that you need? |
| **Research question 2** | ***To describe and analyse the cultural beliefs and linguistic challenges faced by refugees from African nations, and the extent to which primary health care organizations and providers provide services that respond to these challenges*** |
| **Interview questions** | 5. What are the most important cultural factors that you look for in a health service?  (Prompts: meaning of health, preferred treatment options, provider’s cultural competency, culturally safe environment, cultural expectations, values, beliefs, linguistic issues, respect for and interest in questions and concerns, racism, discrimination)  6. How have your experiences of receiving health care in Australia met your needs or expectations in these ways?  (Prompts: how the service meet personal understanding of health, preferred treatment options, cultural expectations, values, beliefs, linguistic issues, respect for and interest in your questions and concerns, what should be done differently to make service responsive to cultural needs)  7. Do you have any other comments about how services meet your cultural needs or expectations? |
| **Exit questions** | 8. Do you have any other comments or additional information? Or questions?  9. Who else do you think I can talk to? |
