# Supplementary material for: Responding to health literacy of refugees in Australian primary health care settings: a qualitative study of barriers and potential solutions
Source: BMC Health Serv Res. 2024 Jun 21;24:757. doi: 10.1186/s12913-024-11192-9 (PMC11193211; doi:10.1186/s12913-024-11192-9)
Supplement: Supplementary file 2 — Supplementary Material 2. [file 12913_2024_11192_MOESM2_ESM.docx]

|  | **Interview Schedule** |
| --- | --- |
| ***Introduction*** | My name is Prince Peprah and I come from Ghana. I arrived in Australia in March 2020 (a week before the international travel ban) to do my PhD at UNSW—mix feelings and experiences due to COVID-19. Thank you very much for agreeing to participate in this interview. Sharing your experiences with us will help the researchers to understand how to support primary healthcare organizations to be more responsive to people from refugee backgrounds. We would like the interview to be a conversation, we really want to hear about your experiences accessing primary healthcare in Australia by people from refugee backgrounds.  Please note:   - You can withdraw from the interview at any time. The interview is confidential   Are you happy for the interview to be audio-recorded? This will allow me to concentrate on the discussion and not focus on taking notes.  The interview will be transcribed and de-identified.  Do you have any questions before we proceed? |
| **Basic details** | 1. Can you please tell me a little about yourself?  (Prompts: Professional background, gender, age, educational attainment, number of years working in primary healthcare, number of years in current position, category of work) |
| **Research question 1** | ***To examine how primary health care organizations and providers identify and support health literacy needs of refugees from African nations*** |
| **Interview questions** | 2. To start off, can you please tell me about your experiences of providing primary health care to people from refugee backgrounds?  (Prompts: what works/what doesn’t, importance, main challenges)  3. In your experience, what are the main challenges that people from refugee background face in accessing primary health care in Australia?  (Prompts: appointment booking, finding the appropriate service, understanding of treatment and health information, communication and interaction, culturally-appropriate service, language issues of people from refugee background, how the challenges are identified)  4. What strategies might be used to overcome barriers people from refugee backgrounds face accessing primary health care such as…..? (Prompts: current strategies, what involves in the strategies, how the strategies are implemented, and effectiveness of the strategies) |
| **Research question 2** | ***To describe and analyse the cultural beliefs and linguistic challenges faced by refugees from African nations, and the extent to which primary health care organizations and providers provide services that respond to these challenges*** |
| **Interview questions** | 5. Do you believe culture and language is important in people from refugee background’s health care?  (Discuss why and previous examples/encounters)  5a. What do you consider as features of health service that meet the cultural and language needs of people from refugee background?  Prompts: (discuss evidence of what works/what doesn’t, whether or not services meet what participant described)  6. Could you please tell me about the current approaches/strategies you use to deliver health care that meet the cultural needs of people from refugee backgrounds? |
| **Research question 3** | ***To investigate the barriers and enablers to cultural, linguistic and health literacy responsiveness*** |
| **Interview questions** | 7. What do you think help/enable you to address problems that people from refugee background face in using health care such as…?  **Hint:** ***Health literacy*** *means asset, skill and competence that one can develop across life course to make informed decisions about health in order to promote and maintain good health and increase quality of life*. ***Cultural and linguistic responsiveness*** *refers to health care services that are respectful of, and appropriate to, the distinct sociocultural, linguistic needs, health beliefs and practices of populations and communities*. (Discuss enablers to health literacy, cultural and linguistic responsiveness)  8. What do you think are the major challenges in providing services that meet the needs of people from refugee background such as…..?  (Discuss challenges to addressing health literacy, cultural and linguistic needs)  9. What strategies do you think could be used to improve the responsiveness of primary health care services to people from refugee background?  (Discuss strategies to improve health literacy, cultural and linguistic responsiveness) |
| **Exit questions** | 10. Do you have any other comments or additional information? Or questions?  11. Who else do you think I can talk to? |
